# Influence of Different Types of Cemented Carbide Blades and Coating Thickness on Structure and Properties of TiN/AlTiN and TiAlN/a-C:N Coatings Deposited by PVD Techniques for Machining of Wood-Based Materials

**DOI:** 10.3390/ma14112740

**Published:** 2021-05-22

**Authors:** Beata Kucharska, Jerzy Robert Sobiecki, Pawel Czarniak, Karol Szymanowski, Konrad Cymerman, Dorota Moszczyńska, Peter Panjan

**Affiliations:** 1 Faculty of Materials Science and Engineering, Warsaw University of Technology, Woloska 141, 02-507 Warsaw, Poland; robert.sobiecki@pw.edu.pl (J.R.S.); Konrad.Cymerman.dokt@pw.edu.pl (K.C.); dorota.moszczynska@pw.edu.pl (D.M.); 2 Department of Mechanical Processing of Wood, Institute of Wood Science and Furniture, Warsaw University of Live Science, Nowoursynowska 159, 02-787 Warsaw, Poland; pawel_czarniak@sggw.pl (P.C.); karol_szymanowski@sggw.pl (K.S.); 3 Department of Thin Films and Surfaces, Jožef Stefan Institute, Jamova 39, SI-1000 Ljubljana, Slovenia; peter.panjan@ijs.si

**Keywords:** PVD method, AlTiN, a-C:N coatings, tool durability tests, wood machining

## Abstract

The influence of different types of cemented carbide blades and thickness of TiAlN/a-C:N and TiN/AlTiN protective coatings used in the wood industry on cutting performance has been studied. Three types of WC-Co cemented carbide blades with different cobalt content were used in the study. The thicknesses of both types of coatings were ~2 and ~5 µm. The structure, chemical and phase composition were studied using transmission and scanning electron microscopy (TEM, SEM), X-ray dispersion spectroscopy (EDX) and X-ray diffraction (XRD), respectively. The adhesion was evaluated by scratch test. Nanohardness and durability tests of uncoated and coated blades were performed. We found that the blades covered with 5 µm TiN/AlTiN coatings exhibited the best durability characteristic. The cutting distances were within the range ~6700-~7080 depending on the substrates in comparison with pure substrates (~4300–~4900) and 2 µm TiN/AlTiN coatings (~5400–~6600). The presence of a thin and soft outer a-C:N layer aggravates the nanohardness and durability of the coated blades.

## 1. Introduction

Development in the field of tool wear protection is made possible by new types of advanced protective coatings. Over 90% of all cemented carbide tools are coated using chemical vapor deposition (CVD) and physical vapor deposition (PVD) [1,2,3,4]. The protective coating suitable for advanced applications of cutting tools must endure extremely high thermo-mechanical loads and resist to degradation in severe environments. Coatings that withstand such demanding conditions well are often aluminum-based e.g., TiAlN, AlTiN, CrAlN, Zr-ZrN-(Zr,Al,Si)N [2,3,4].

Carbon-based coatings (e.g., diamond like carbon—DLC) also play an important role in many areas of industrial applications. The most common is a-C:X, where carbon can be enriched with nitrogen or another element. This type of coating is characterized by very good tribological properties, as demonstrated by Pancielejko et al. [5,6]. The authors mentioned above used this kind of tool coatings for laminated MDF (medium-density fiberboard), pinewood slats, and floorboards milling. According to Precht et al. [7], the friction coefficient of DLC films is 0.2. Moreover, Faga and Settineri used RF PACVD (radio frequency plasma assisted chemical vapor deposition) and cathodic arc evaporation method to deposit DLC coatings on the high speed steel HSS 18 and alloy steel AS 90CMV8 [8]. An even better way to improve the durability of coatings is to prepare them in the form of nanocomposites [9]. This solution encompasses advantages of both the concepts of carbon-based nanocomposite coatings and those of multilayer coatings. New nanoscale single-layer composite coatings composed of coexisting metastable hard phases like fcc (Ti,Al)(N,C) and amorphous carbon a-C.

Sheikh-Ahmad and Morita [10] or Sheikh-Ahmad et al. [11] found that in the case of coated cemented carbide tools (extra fine carbide grain, low Co content) with very sharp cutting edge, a problem arises with coating adhesion and unfavorable residual stress in the coating which cannot be released due to significant hardness of the substrate. This means that it is not only important to use advanced protective coatings, but also to optimize the substrate surface preparation and parameters of coating deposition. The authors suggest that the use of cobalt etching or tungsten carbide chemical etching increase the adhesion of the coating. Castanho and Vieira improved the durability of TiAlN thin coatings by using interlayers of ductile metal, without causing a disruption in the mechanical properties [12]. Three ductile metals were used as interlayers: aluminum, titanium and copper. In Beer et al. [13] or Faga and Settineri [8] papers, tools were subjected to duplex modification, first by low temperature ion nitriding, followed by deposition of hard coatings Cr_2_N. Gilewicz et al. [14] proposed the chromium sublayer 0.1 µm in order to improve adhesion between substrate and coating. Pancielejko et al. [5] used a chromium adhesive sublayer with total thickness of 0.6 µm, W/W-DLC interlayer and W-DLC layer with glass-like structure. In the work of Pancielejko et al. [6], the Cr interlayer (0.3 µm) was deposited on the planer knives. Warcholiński et al. [15] applied TiAlN/TiN and CrCN/CrN multilayers with interlayers: for titanium-based coatings, Ti (0.1 µm) + TiN (0.2 µm) and for chromium based coatings, Cr(0.1 µm) + CrN (0.2 µm).

Studies of the influence of substrate hardness on mechanical and tribological properties of Cr-based coatings, both in the form of CrN and Cr_2_N applied to tungsten carbide blades intended for processing of wood materials, were carried out by Noveau et al. [16]. Their research showed that coatings hardness is higher than substrate hardness only for softer tool materials. This results from the fact that during the deposition process, compressive stresses are generated within the coating. The lower the substrate hardness and thus the E value, the higher the internal stress that can be released in the substrate. For harder substrates, the microhardness of the coating reaches its highest value thanks to the support provided by the substrate. However, harder coatings have in turn provided greater resistance to abrasion and adhesion. 

The deformation mechanisms of TiN coating thickness deposited on the same type of substrate have been quite well studied by Ma et al. [17]. The authors focused on mechanical properties, especially on the coating deformation of 0.7 (cathodic arc evaporation—CAE), 2, 3.7 and 4 µm (low voltage electron beam evaporation—LVEB) thick TiN coatings. They found that for thinner coatings, shear stress occurring at the grain boundaries along columns play a dominant role. For thicker coatings, internal cracking prevails. The authors stress the important role of both grain structure and coating thickness. It was revealed that an increase in coating thickness also causes an increase in coating hardness because of the influence of the softer substrate. It was noted that even at low loads, there was a strong influence from the substrate on the hardness. At the same time, the thickness increase was accompanied by a rise in inter-columnar tensile stress from 2.2 GPa for 0.7 µm up to 3.55 GPa for 4 µm.

Vereschak et al. [18] analyzed TiN-(Ti,Al,Si)N coatings with thicknesses of 2.0, 3.5, 5.0, 7.0, 11.0 and 15.0 µm and they found that an optimal coating thickness exists for each of the different coating structures, however, it is assumed that in the case of single coatings, this range is within 2–10 µm. They also observed a sequence of internal delaminations in the case of thicker coatings. Moreover, with increasing thickness, internal microcracks appear.

In the case of multilayer coatings, each of the individual layers has a different chemical composition, the microstructure of each such layer is extremely important. The proportions between the components within a single layer determine to a large extent the properties of the entire coating. The study by Lin et al. [19] focused on a multilayer coating consisting of CrN/AlN bilayers. In this study, the atomic ratio Al/(Cr + Al) was increased from 19.1 to 68.7% by decreasing the Cr target power from 1200 to 200 W. The mentioned above changes of parameters deposition caused a decrease in the bilayer period from 12.4 to 3 nm. The hardness of the coating reached its maximum at an Al/(Cr + Al) ratio equal to 59.3%. In result, the reduction of CrN thickness, makes an internal stress lower, which can be attributed to an increase Al content and increase of the total number of interlayers. The reduction of bilayer thickness (CrN and AlN thicknesses within single bilayer were 1.5 nm and 2.5 nm, respectively) led to an increase of hardness up to 42 GPa, a decrease in the friction coefficient and low wear rate.

Seidl et al. [20] analyzed the effect of the number of bilayers in the (TiAlTa)N/(AlCr)N coating. At a fixed bilayer period of 24 nm, the total thickness of the coating was 0.8, 1.6, 2.4, 4.8 and 16 µm. As substrates, they used silicon wafers, alumina, hard metal and austenitic stainless steel. It turned out that only thin coatings (up to 4 µm in thickness) show a clear relationship between stress and thickness, while all coatings above 4 µm exhibit similar stress levels. As a result, the microhardness of the layer increased slightly to a layer thickness of about 4 µm, and above this thickness, it was constant. 

In terms of the mechanical properties of a coating, the limit thickness at which came up unbeneficial changes of the phase variant that directly affects its strength is also relevant. This problem concerns layers containing AlN, which can occur both in metastable cubic form (c) and as stable wurtzite (w), which is characterized by inferior mechanical properties. Determination of the optimal thickness in this respect can be found in the work by Chawla et al. [21] in which an AlN/TiAlN multilayer coating was tested. It turned out that the cubic phase form can be stabilized at a thickness of c-AlN up to 17 nm. The authors showed also that Ti_0.35_Al_0.65_N is the maximum Al content in super-saturated cubic Ti_1−x_Al_x_N that can be used as the underlaying layer below AlN to stabilize the cubic AlN phase.

The influence of coating thickness is also visible in the thermal properties of coatings containing aluminum. Tlili et al. [22] provided information on the influence of thickness on thermal properties for three different types of multilayer coatings i.e., Cr/CrN/CrAlN, CrN/CrAlN and Cr/CrN. The studies show that the thermal conductivity of single coatings is higher or equal than that of multilayer coatings. Generally speaking, coatings consisting of a large number of intermediate layers showed a decrease in thermal conductivity. 

A large amount of literature discusses coatings deposited on tools for machining of metallic materials. However, the processing of wood-based materials is more complicated because they have extremely specific properties resulting from the complex chemical composition, heterogeneous structure and structure anisotropy. They are porous materials with a fibrous structure and a strong anisotropy of physical and mechanical properties depending on the anatomical direction. Due to the three-dimensional anisotropy of the wood, the cutting conditions vary considerably, depending on the position of the wood relative to the tool during cutting. 

Due to such a variety of properties, wood and its derivatives are extremely difficult materials to process. Due to these material characteristics of the material, the wear processes of the cutting blades take place in a completely different, often more drastic way than in the case of metal processing. This fact is not obvious considering the much higher values of the mechanical properties of metals in comparison to wood and its derivatives. In the case of wood-like materials such as particle board, there is also a contamination factor. Particle board is a construction material made of wood chips pressed in a resin under pressure and at high temperature, which are most often post-production waste. This allows for the penetration of impurities into the workpiece material, the most undesirable of which is sand, which destroys machining tools [23]. All these factors make the wood and its derivatives machining still a challenge for modern tools. Due to the complexity and variety of processes taking place during the cutting of this group of materials, the wear mechanisms are different than in the case of metalworking [24]. Composites are also difficult to machine because they are anisotropic and non-homogeneous. Additionally, their reinforcement phase could be abrasive and damage the tool [25]. Among the variety of cutting tools used in machining of composites are: high-speed steel (HSS), tungsten carbide, cubic boron nitride (cBN) and polycrystalline diamond (PCD). In the case of composites, apart from the tool material itself, the processing parameters are also very important [26].

Protective coatings for sintered tungsten carbide tools for machining wood-based materials are made primarily by PVD methods. Most often, PVD coatings based on nitrides, mainly CrN and TiN (both single and multi-layer, e.g., CrN/CrCN or TiAlN), were tested.

Djouadi et al. produced CrN or Cr_2_N coatings on high-speed steel and sintered carbide by the PVD method [27]. These coatings resulted in a two-fold decrease of tool wear when machining MDF. In turn, Gilewicz et al. examined the CrCN/CrN and CrCN/CrN + ta-CN coatings produced on HS18-0-1 steel [28]. The workpiece material was dry pine wood. It has been shown that the wear of tools with CrCN/CrN coatings was about two times lower than tools without the coating, and the formation of the ta-C coating on the surface allowed for a 15% increase in durability.

Similar CrN/CrCN coatings were deposited on M2 high-speed steel and cemented carbides by the arc evaporation method [29]. The results of wood-like materials machining have shown that CrN/CrCN multi-component coatings increase the tool life of the high-speed steel by 33% in comparison to the CrN coating and 170% with respect to the uncoated tool. In the case of cemented carbides, these values were 7% and 110%, respectively. In the work of Kuleshov et al., the ZrN and Mo + Mo_2_N coatings were studied [30]. They were subjected to the powder sulfur nitrocarburization process with NH_2_-CS-NH_2_. The sulfur nitro-carbonation process resulted in a further increase in durability by about 20%. The work of Kazlauskas et al. concerns CrN and TiAlN coatings on cemented carbides with a thickness of about 2 µm, also produced by the PVD method at temperatures of 700 °C for CrN and 800 °C for TiAlN, respectively [31]. The processed material was solid oak wood, which was subjected to a milling process. It was shown that the CrN coating was characterized by better properties as the rake face wear was the lowest and amounted to 4 µm, while for the TiAlN coating, it was 11 µm.

Since, in the field of woodworking tools, the influence of microstructure or thickness of a coating on its properties has been examined practically only for Cr-based coatings, it was necessary to examine this issue also for nanolayer TiN/AlTiN or double TiAlN/a-C:N coatings which can be used in the woodworking industry. Hence, there is a need for a comprehensive analysis of the relation of this coating’s microstructure, phase composition, thickness, microhardness and durability. As it follows from the above-mentioned overview, there is also a lack of comprehensive knowledge about the influence of the kind of substrate (e.g., the cobalt content in cemented carbide) on the properties of the nanolayer TiN/AlTiN and double layer TiAlN/a-C:N coatings. 

In this study, the influence of the substrate type differing in the cobalt content and the thickness of the coatings on the microstructure and properties of TiAlN/a-C:N and TiN/AlTiN coatings was obtained by magnetron sputtering.

## 2. Materials and Methods

### 2.1. Coating Preparation

The studies described in this paper included TiN/AlTiN and TiAlN/a-C:N coatings deposited on cemented carbide blades. Three types of WC-Co cemented blades with different cobalt content (low, medium, high) were used in this study: Co-L-4.5 wt.% Co-M-5.6 wt.% and Co-H-7.5 wt.% The dimensions of the cutting tools (manufactured by FABA) amounted to 30 mm × 12 mm × 1.5 mm. 

**Figure 1:**
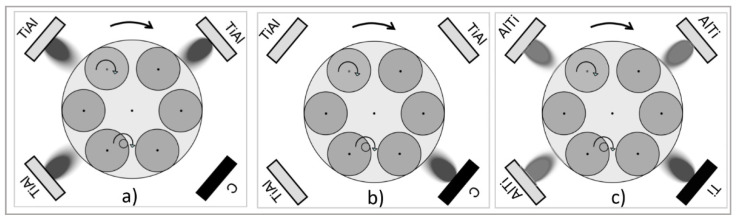
Configurations of targets for the deposition of a TiAlN/a-C:N coating bottom TiAlN layer (**a**), TiAlN/a-C:N coating top a-C:N layer (**b**) and AlTiN/TiN coating (**c**) [32].

Both coatings were deposited by an industrial direct current (DC) magnetron sputtering system (Figure 1a–c). The deposition system was equipped with four rectangular unbalanced planar magnetron sources arranged in the corners of a chamber.

The blades were either in horizontal position (with 3-fold rotation, designation 3L) or in vertical position (with 2-fold rotation, designation 2P). In the first case, the thickness of both kinds of coatings was ca. 2 µm, while in the latter case, it was ca. 5 µm. In the deposition chamber, the substrates were heated to 400 °C for 60 min. Mid-frequency ion etching with pulsed bias on a turntable at a voltage of 650 V and a frequency of 240 kHz was conducted for 45 min in a mixed argon (flow rate 180 sccm) and krypton (flow rate 50 sccm) atmosphere under a pressure of 0.35 Pa. 

After loading, the vacuum chamber was evacuated to a base pressure of 3.0 × 10^−3^ Pa. The total operating pressure was maintained at 0.6 Pa, with flow rates of nitrogen, argon and krypton of 100, 160 and 110 mL/min, respectively. TiN/AlTiN coatings were deposited from three segmental Ti–Al targets and one Ti target (dimensions 88 × 500 mm). The Ti–Al targets had 48 cylindrical Al plugs embedded into the racetrack in order to produce the coatings at an approximately 1:2 atomic ratio of Ti:Al. That is why the AlTiN name is used in the contrary of the TiAlN name where the amount of Ti:Al in the target was 1:1. The periodicity of the layer structure (the number of layers that repeat in the layer structure) was determined by substrate rotation. The thickness of the individual TiN and AlTiN layers was determined from the deposition time of individual targets and the rotation speed of the turntable and was in the range of 5 nm for the TiN layer and 30 nm for the AlTiN layer. The power on each of the Ti–Al and Ti targets amounted to 9.5 and 4 kW, respectively.

In the case of TiAlN/a-C:N coatings, three cathodes were used for the deposition of TiAlN and only one for the deposition of the a-C:N top layer. Target materials consisted of TiAl and pyrolytic graphite (purity 99.8%). In the first part, three cathodes with segmental TiAl targets of an approximately 1:1 Ti to Al ratio were active for depositing TiAlN.

The flow rate of nitrogen was 170 sccm and the power on each of the TiAl targets was 9.5 kW. In the second part, one graphite target was active with a power of 4 kW and a nitrogen flow of 70 sccm. The process pressure during deposition was constant at around 0.4 Pa. The total thickness of the TiAlN/a-C:N coating was 4.9 μm (for 3-fold rotation). The thickness ratio of TiAlN to a-C:Nx was 9:1.

### 2.2. Characterization of Coatings

Analysis of the morphology, surface topography, and chemical composition of the coatings was carried out using Hitachi SU-70 and S-3500N scanning electron microscopes (SEM) equipped with a Noran EDX (energy-dispersive X-ray spectroscopy) microanalysis system. Transmission electron microscope (TEM) JEOL JEM 1200 was applied for structural characterization of the TiN/AlTiN and TiAlN/a-C:N coatings. The thickness of the coatings was estimated based on metallographic cross-sections also using SEM techniques. The surface roughness measurements were conducted on the surface of the samples using a Mitutoyo SJ210 Surface Roughness Tester. The stylus tip was moved along the coated and uncoated surfaces with a cut-off length of 0.8 mm. The average roughness parameters (the average value of three measurements) (R_a_) were used to evaluate the surface quality of the specimens. The XRD phase composition of the obtained layers was determined with a Bruker AXS D8 Discover X-ray diffractometer using CuK_α_ radiation with a wavelength of λ = 0.154 nm. In order to provide the opportunity to probe the structural evolution of solid near the surface, the grazing incidence X-ray diffraction (GIXRD) technique was used. A monochromatic X-ray beam with a wavelength of 0.15 nm was used. The GIXRD setup is equipped with a parabolic Göbel mirror and a conventional line focus Cu radiation tube (40 kV/40 mA). The incidence angle was fixed 2° with 2θ range 20–120° with a step of 0.025°.

To obtain a complete characterization of examined samples, additional XRD tests were performed for the crystalline size determination. The Scherrer equation (or the Debye–Scherrer equation) has been applied [33]. The Scherrer equation (1) relates the width of a powder diffraction peak to the average dimensions of crystallites in a polycrystalline sample:

(1) $$\beta \left(2\theta \right)=\frac{K\lambda }{\mathrm{Lcos}\theta },$$

where β(2θ) is the crystallite size contribution to the peak width, *K* is a constant near unity and L is the average size of the crystal in a direction normal to the diffracting plane (h k l).

The thickness of the coatings was determined by the ball cratering technique (Calotest) with the use of optical microscopy (OM) described in work of Wróbel-Knysak et al. [34]. The used ball was made of 100Cr6 steel, its diameter was Ø = 30 mm while its rotation speed 7.5 rev/s. Sphere rotation time was determined by optimum size of crater and set on 200 to 400 s. The abrasive paste used in tests was diamond-based paste with 0.25 µm average grain size.

Adhesion of the coatings to the substrates was analyzed using a CSM Instruments RST scratch tester with diamond stylus with a 200 µm spherical tip radius. The indenter load increased from 1 to 50 N over a 5 mm length. The adhesion of the coatings was evaluated on the basis of the L_C3_ value. The L_C3_ critical load occurs with a coating delamination force and was determined based on an acoustic emission measurements and post-test scratch images.

Hardness of the different substrates and coatings was determined by means of nanoindentation using the NanoTest Vantage (Micromaterials Ltd., Wrexham, UK) with a diamond Berkovich indenter. The mean hardness was obtained from a minimum of 6 indentations. The indentation load was 20 mN and loading and unloading time 20 s.

### 2.3. Durability Tests

The durability tests were conducted on a Busellato JET 130 standard working center at the Warsaw University of Life Sciences. The spindle’s rotational speed and feed speed was set to 18,000 rpm and 2.7 m/min, respectively, which provides a feed rate of fz = 0.15 mm per tooth, The diameter of the head produced by the company FABA equipped with one replaceable blade was 40 mm. For each variant, 4 blades were used (overall 8 cutting edges). During statistical processing of the data, the extreme values, i.e., the minimum and maximum value for a given variant, were considered unrepresentative and rejected. The more detailed information about this test was presented in the authors’ previous work [35].

After each pass (feed distance 0.7 m), the tool wear was measured on a workshop optical microscope. The tool wear indicator (VB_max_) that showed the end of the blunting procedure was chosen as 0.2 mm. The feed distance was calculated into cutting distance according to Equation (2):

(2) $$L_{t}=\frac{V_{C}\frac{L}{V_{T}}}{2}=\frac{\pi Dn}{2}\cdot \frac{L}{V_{T}},$$

where:

*D*—iameter of tool [m];
*n*—rotational spindle speed [1/min];
*V_T_*—feed speed [m/min];
*V_C_*—cutting speed [m/min];
*L*—feed distance [m/min];
*L_t_*—cutting distance.

A three-layer standard chipboard with a nominal thickness of 18 mm was used in the blunting procedure. The mechanical and physical properties of the workpiece material are shown in the Table 1 [36,37,38,39,40,41,42,43]. The procedure of detailed durability has been presented in the authors’ previous paper [35].

## 3. Results

### 3.1. XRD and TEM Analysis

**Figure 2:**
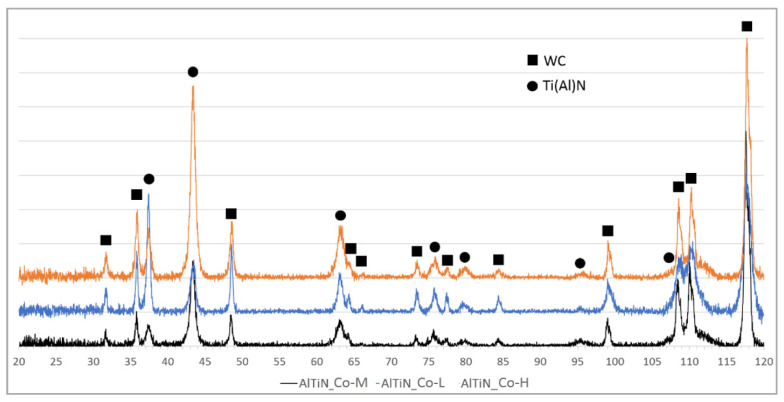
XRD analysis graph of 5 µm thick TiN/AlTiN coating deposited on three different cemented carbide substrates: Co-L, Co-M and Co-H.

**Figure 3:**
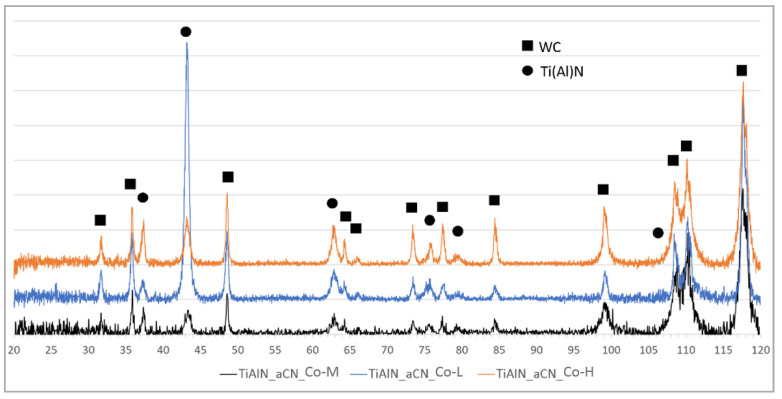
XRD analysis graph of 5 µm thick TiAlN/a-C:N coating deposited on three different cemented carbide substrates: Co-L, Co-M and Co-H.

The results of XRD phase composition analysis of TiN/AlTiN and TiAlN/a-C:N coatings of 5 µm thickness deposited on different substrates are shown in Figure 2 and Figure 3. All X-ray spectra for each variant of coatings show the same phase composition regardless of cobalt content. The phases identified in samples by GIXRD analysis were WC (substrate), TiN and TiAlN (both from hard coating). The results of diffraction tests for coatings with a thickness of 2 µm did not differ from the spectra obtained for thicker coatings.

The size of crystallites was calculated by the Scherrer method for the Ti(Al)N phase for both coatings. The results of calculations are presented in Table 2. The obtained data indicate no influence of used WC-Co substrate on the size of crystallites. It was noticed that the smaller crystallite size was obtained for the TiN/AlTiN coating—about 10 nm. It can be related to the sandwich-like structure of these coatings. However, for the TiAlN/a-C:N layers calculated, the crystallite size was about 25 nm, which is an increase of about 150% compared to the TiN/AlTiN layers. This coating is a double layer coating.

**Figure 4:**
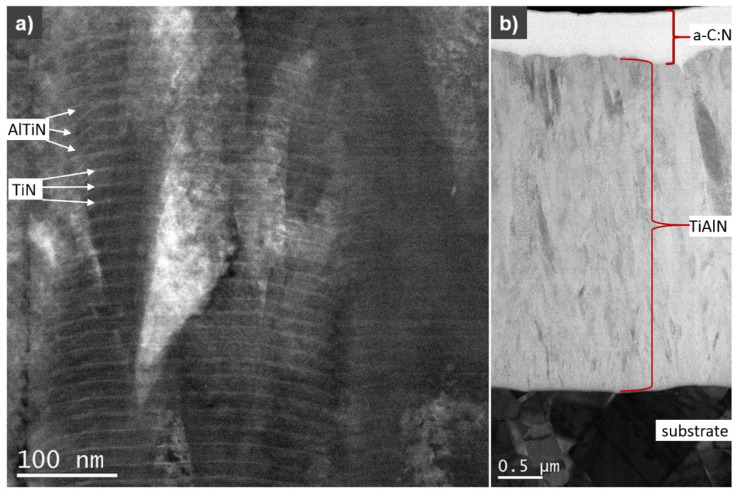
A TEM image of cross-section of a TiN/AlTiN 5 µm thick coating (**a**) and TiAlN/a-C:N 5 µm thick coating (**b**) obtained on the Co-M substrate.

Figure 4 shows a cross-section image of a TiN/AlTiN coating obtained on the Co-M substrate. It has a columnar microstructure and it consists of multilayers. The TiN layer, visible as a brighter zone, is about 6 times smaller in thickness with a darker zone of AlTiN. These results confirm the assumption that the thickness ratio of individual TiN layers is about 5 nanometers and the AlTiN layer is about 30 nanometers, related to the evaporation time of individual targets and the rotation speed of the turntable. That is why the TiN/AlTiN coating may be called the nanolayer coating. The thickness of the outer a-C:N layer (Figure 4b) is within the range 500–590 nm.

### 3.2. Microstructure, Chemical Composition and Roughness of Coatings

**Figure 5:**
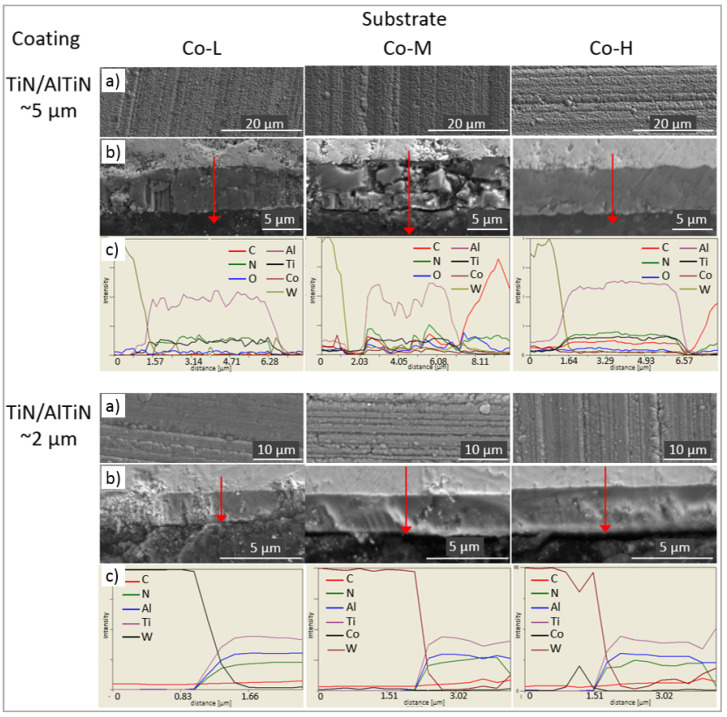
Morphology (**a**), metallographic cross-section SEM images (**b**) and EDX line scan composition profiles (**c**) of TiN/AlTiN nanolayer coatings deposited on Co-L, Co-M and Co-H substrates.

**Figure 6:**
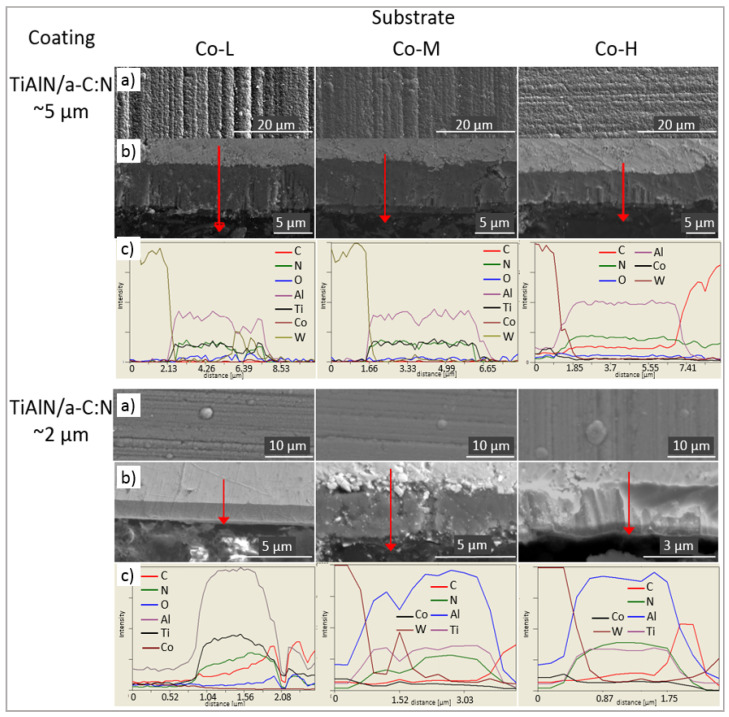
Morphology (**a**), metallographic cross-section SEM images (**b**) and EDX line scan composition profiles (**c**) of TiAlN/a-C:N double layer coatings deposited on Co-L, Co-M and Co-H substrates.

Figure 5a and Figure 6a show the surface morphology of TiN/AlTiN and TiAlN/a-C:N coatings deposited on three kinds of cemented carbide blades: Co-L, Co-M and Co-H. Characteristic globules and groove traces originally appearing on the substrate are observed on the surface of all the coatings. On the surface of the nanolayer, the TiN/AlTiN coating deposited on the Co-H substrate, a few defects such as cavities and nodules are visible. On all coating surfaces, numerous globules appear.

Based on fracture cross-section SEM images, it can be concluded that all coatings demonstrate good adhesion to the substrate. The TiAlN/a-C:N coating, due to its high hardness and brittleness, cracked in some places during grinding and polishing, as shown in Figure 6. The chemical composition on the metallographic cross-section of the TiAlN/a-C:N coating also varies. Such oscillation of composition could also be the result of the rough surface on the coating cross section. The thickness of the layers is in accordance with the assumed process parameters and is about 5 micrometers for samples placed in the horizontal position and about 2 micrometers for samples placed in a perpendicular position to the targets. The outer a-C:N layer is visible in the pictures. Its thickness is about 500 nm and is in accordance with the process parameters which assumed that depending on the target deposition times, the ratio of the a-C:N to TiAlN layer thickness was 1:9. 

The surface roughness of the bare and coated substrates is shown in Table 3. The Co-H substrate (with the highest cobalt content) shows the lowest R_a_, and conversely, the Co-L substrate has the highest roughness. In all the analyzed cases, coatings deposited on the Co-H substrate are characterized by the lowest R_a_ (regardless of their thickness). This may be due to the use of the substrates with the lowest roughness.

It is worth noting that the roughness of the TiAlN/a-C:N coating deposited on all substrates S, both with a thickness of ca. 5 µm and ca. 2 µm expressed in the R_a_ parameter, was lower than the roughness of the substrates itself. This unusual phenomenon can be explained by the amorphous nature of the a-C:N top layer. The results received by Czarniak et.al [40] obtained with the TEM technique for the same double TiAlN/a-C:N coating confirmed existence of such kind of layer structure.

During analysis of the nanolayer AlTiN-type coatings, it was observed that for all substrates and coating thicknesses (ca. 5 µm and ca. 2 µm), the surface roughness of the substrate is lower than that of the coating surface. For example, the R_a_ value for the Co-L substrate and AlTiN coatings of ca. 5 µm and ca. 2 µm applied to this substrate are 0.150, 0.163 and 0.184 µm, respectively. Even greater differences between the roughness of the substrate and the roughness of the coating were apparent for Co-M and Co-H substrates. This coatings performance confirms the general opinion that the roughness of the coated substrate is always higher than the roughness of the bare substrate. The reasons for this are twofold: (a) the roughness of the substrate increases after ion etching, which is a standard in situ cleaning technique; (b) the roughness of the coated substrate increases due to the formation of growth defects (nodular defects, flakes, pinholes, craters).

### 3.3. The Calotest Results

**Figure 7:**
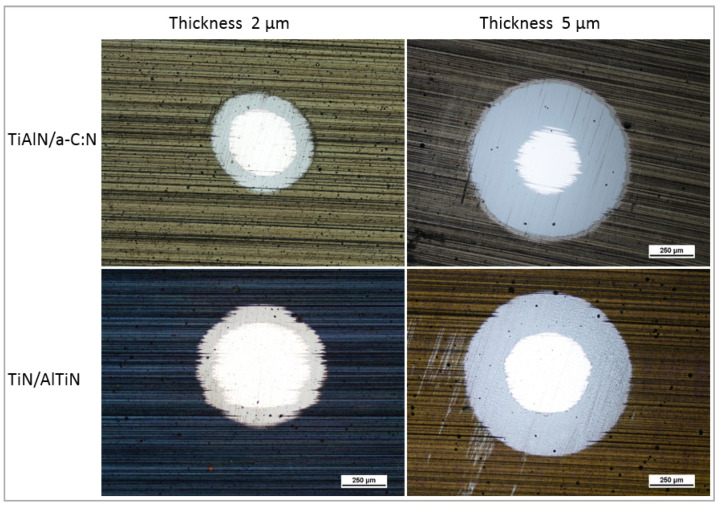
OM image of ball crater through the TiN/AlTiN and TiAlN/a-C:N coatings with the thickness of 2 and 5 µm, respectively.

The results of the calculated coating thickness by Calotests are given in Table 4. The calculations were carried out on the basis of the crater diameter measurements (Figure 7). It can be seen that the produced layers have a thickness similar to the assumed thickness. In the case of 2 µm layers, a slightly smaller thickness is noticeable, while in the case of 5 um layers, the thickness is higher than assumed. However, the high roughness affects the measurement accuracy. It has to be noticed that the Calotest confirmed the presence of a thin layer on the TiAlN/a-C:N coatings. Figure 7 shows the selected OM images of the ground section from which the coating thickness was determined.

### 3.4. Adhesion

**Figure 8:**
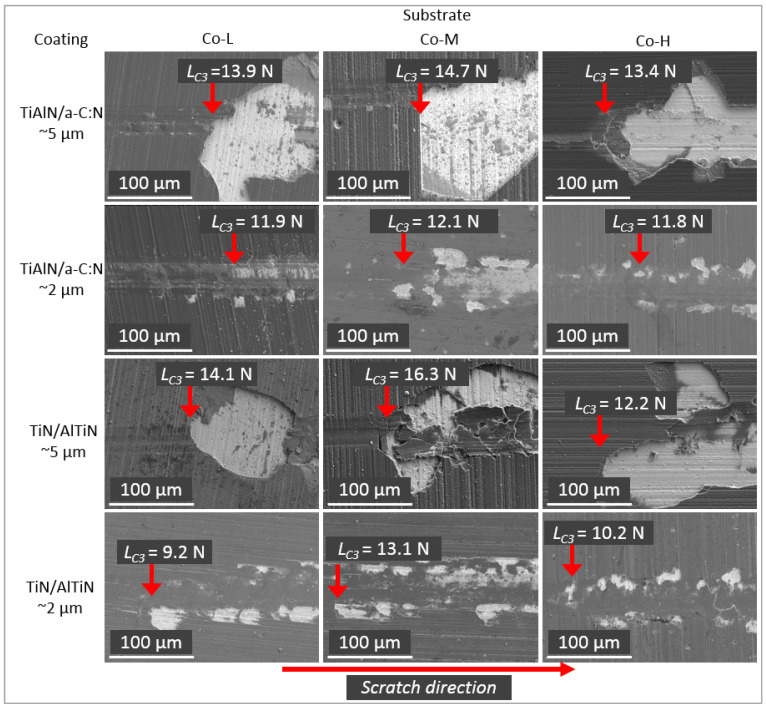
SEM images of the scratch tracks at the position of the critical load *L*_*C*3_ of the nanolayer TiN/AlTiN and TiAlN/a-C:N double layer coating deposited on Co-L, Co-M and Co-H substrates.

In order to analyze the adhesion of the nanolayer TiN/AlTiN and TiAlN/a-C:N coatings to the Co-L, Co-M and Co-H substrates, a scratch test was performed and the critical load (*L*_*C*3_) was evaluated (Table 5). It is observed that in the case of all thicker 5 µm coatings (TiN/AlTiN and TiAlN/a-C:N), the interfacial decohesion and the embrittlement of the layer occurred (Figure 8). The significant decohesion and pure adhesive fracture took place irrespective of the kind of substrate. In the case of thicker coatings, the lowest critical load *L*_*C*3_ was obtained for the coatings deposited on the Co-H substrate.

In the case of the thinner 2 µm coatings, the values of critical load were lower, but at the same time, the coating failure were less extensive.

### 3.5. Nanohardness

The results of nanohardness tests indicate that the cemented carbide, which is characterized by the highest cobalt content (Co-H), has the lowest nanohardness (Table 6). On the other hand, the TiAlN/a-C:N and TiN/AlTiN coatings produced on all three types of carbides have lower nanohardness values compared to the substrate. TiAlN/a-C:N and TiN/AlTiN coatings with a thickness of 2 µm have higher nanohardness values compared to their 5 µm counterparts. This can be explained by the influence of a harder substrate on the final result of the nanohardness of the coating-substrate system. The presence of a thin amorphous a-C:N layer causes a significant reduction of nanohardness compared to the TiN/AlTiN coating for both 2 and 5 µm thick coatings. 

### 3.6. Durability Tests

Analysis of durability test results (Table 7) shows significant differences in the behavior of a tool coated with an TiN/AlTiN nanolayer coating and a TiAlN/a-C:N coating. In the case of the nanolayer TiN/AlTiN coating with a thickness of approx. 5 µm, a clear improvement of tool lifetime was observed for all the variants of the WC-Co tungsten carbide substrate. The reduction of the coating thickness to approx. 2 µm makes the modification of the tool not so high.

The results of durability tests do not reflect the results of the nanohardness tests. It is evident that the harder 2 µm thick coatings are less durable compared to 5 µm thick coatings. This can be explained by the insufficient thickness of the coatings (2 µm), which wear out faster than thicker coatings (5 µm). On the other hand, a significant reduction in the nanohardness of the TiAlN/a-C:N coatings compared to the TiN/AlTiN coating results in a reduction in their durability. In the case of TiAlN/a-C:N coatings, a similar relationship can be observed as for TiN/AlTiN coatings, where increasing their thickness increases their durability. However, it should be considered that the increase in tool durability of TiAlN/a-C:N coatings was certainly not as high as that shown by the multilayers AlTiN nanolayer coating. A high level of standard deviation is caused by very high heterogeneity of the standard particleboard. Therefore, this kind of wood-based composite is extremely difficult with regards to machinability.

## 4. Conclusions

It was difficult to determine whether surface roughness affects tool durability. In the case of uncoated substrates, the best durability is the substrate with the highest roughness (Co-L), while the one with the lowest roughness (Co-H) has the worst durability. The increase in durability is primarily influenced by the chemical composition of the substrate, not their roughness. 

In the case of all thicker 5 µm coatings, the interfacial decohesion and pure adhesive fracture of the layer occurred.

The best blade durability characteristics were observed for the multilayer TiN/AlTiN nanolayer coating. In the case of thicker coatings, the tool life was improved despite the fact that the adhesion of thicker coatings to the substrate was worse. This may be due to the fact that the coating being too thin (2 µm) does not significantly increase the tool life. The TiAlN/a-C:N coatings with a lower nanohardness compared to TiN/AlTiN coatings have worse durability characteristics. It is caused by the presence of a thin and soft outer layer which has a great influence on blade properties.

## 5. Future Work

Studies have shown that 5 µm multilayer coatings are the most advantageous alternative. As a continuation of the presented studies, it is planned to produce multilayer CrN/TiN coatings with different thickness ratios of individual zones compared to the single-layer Ti_x_Cr_1−x_ coating, where X = 0.5.

## Figures and Tables

**Table 1:** Mechanical-physical properties of machined material.

Property	Value
Density [kg/m^3^]	650
Flexural strength [N/mm^2^]	13.1
Elastic modulus [N/mm^2^]	3200
Strength in pull out of screws test [N/mm]	70.9
Hardness in Brinnel scale [HB]	2.61
Mineral contamination [%]	0.18
Swelling 24 h [%]	25.6
Tensile strength [N/mm^2^]	0.37
Impregnability 24 h [%]	86.5

**Table 2:** Crystallite size of the Ti(Al)N phase for both coatings 5 µm thick.

		Crystallite Size [nm]		
	Substrate	Co-L	Co-M	Co-H
Coating				
TiAlN/a-C:N		25 ± 2.1	25 ± 2.2	22 ± 2.0
TiN/AlTiN		10 ± 1.1	10 ± 1.0	13 ± 1.2

**Table 3:** Surface roughness of substrates, TiN/AlTiN and TiAlN/a-C:N coatings.

Material	Substrate	R_a_ [µm]	SD
Co-L	―	0.150	0.008
Co-M	―	0.105	0.006
Co-H	―	0.098	0.005
TiAlN/aCN ~5 µm	Co-L	0.129	0.006
	Co-M	0.092	0.005
	Co-H	0.091	0.002
TiAlN/aCN ~2 µm	Co-L	0.135	0.005
	Co-M	0.103	0.009
	Co-H	0.093	0.008
TiN/AlTiN ~5 µm	Co-L	0.163	0.018
	Co-M	0.205	0.001
	Co-H	0.136	0.008
TiN/AlTiN ~2 µm	Co-L	0.184	0.014
	Co-M	0.201	0.012
	Co-H	0.135	0.019

**Table 4:** Summary of coating thickness obtained in the ball wear test.

Coating	Substrate	Thickness [µm]
TiAlN/aCN ~5 µm	Co-L	6.2 ± 0.05
	Co-M	5.8 ± 0.00
	Co-H	6.1 ± 0.04
TiAlN/aCN ~2 µm	Co-L	1.6 ± 0.1
	Co-M	1.5 ± 0.1
	Co-H	1.6 ± 0.2
TiN/AlTiN ~5 µm	Co-L	5.3 ± 0.2
	Co-M	5.6 ± 0.01
	Co-H	5.2 ± 0.1
TiN/AlTiN ~2 µm	Co-L	1.8 ± 0.1
	Co-M	1.9 ± 0.2
	Co-H	1.6 ± 0.05

**Table 5:** Critical load *L*_*C*3_ of the nanolayer TiN/AlTiN and TiAlN/a-C:N double layer coatings deposited on Co-L, Co-M and Co-H substrates.

Coating	Substrate	*L*_*c*3_ [N]	SD
TiAlN/aCN ~5 µm	Co-L	13.9	0.049
	Co-M	14.7	0.098
	Co-H	13.4	0.065
TiAlN/aCN ~2 µm	Co-L	11.9	0.336
	Co-M	12.1	0.131
	Co-H	11.8	0.093
TiN/AlTiN ~5 µm	Co-L	14.1	0.049
	Co-M	16.3	0.425
	Co-H	12.2	0.033
TiN/AlTiN ~2 µm	Co-L	9.2	0.485
	Co-M	13.1	0.075
	Co-H	10.2	0.112

**Table 6:** Nanohardness of TiN/AlTiN and TiAlN/a-C:N coatings deposited on different substrates.

Material	Substrate	Nanohardness [GPa]	*SD*
Co-L	―	37.4	3.4
Co-M	―	36.1	4.9
Co-H	―	35.4	5.8
TiAlN/aCN ~5 µm	Co-L	14.4	1.9
	Co-M	15.7	4.2
	Co-H	12.8	1.8
TiAlN/aCN ~2 µm	Co-L	18.5	0.8
	Co-M	16.0	1.8
	Co-H	15.8	3.0
TiN/AlTiN ~5 µm	Co-L	20.5	2.3
	Co-M	19.6	4.5
	Co-H	23.2	6.1
TiN/AlTiN ~2 µm	Co-L	27.2	6.4
	Co-M	24.8	6.4
	Co-H	24.6	5.4

**Table 7:** Durability test results of TiN/AlTiN and TiAlN/a-C:N coatings deposited on different substrates.

Material	Substrate	Cutting Distance [m]	SD
Co-L	―	4909	537
Co-M	―	4769	561
Co-H	―	4301	1265
TiAlN/aCN ~5 µm	Co-L	5751	199
	Co-M	6592	633
	Co-H	5424	1103
TiAlN/aCN ~2 µm	Co-L	5674	206
	Co-M	5238	504
	Co-H	4947	1120
TiN/AlTiN ~5 µm	Co-L	7083	927
	Co-M	6943	898
	Co-H	6733	397
TiN/AlTiN ~2 µm	Co-L	5626	914
	Co-M	5238	504
	Co-H	6241	857
